# Transcriptome and Weighted Gene Co-Expression Network Analysis for Feather Follicle Density in a Chinese Indigenous Breed

**DOI:** 10.3390/ani14010173

**Published:** 2024-01-04

**Authors:** Jiangxian Wang, Wei Wei, Chaohui Xing, Hao Wang, Meng Liu, Jinmei Xu, Xinxin He, Yanan Liu, Xing Guo, Runshen Jiang

**Affiliations:** College of Animal Science and Technology, Anhui Agricultural University, Hefei 230036, China; wangjiangxian12@126.com (J.W.); suhu15@126.com (W.W.); xch1870300782@163.com (C.X.); wanghao20128@126.com (H.W.); chenhong506@163.com (M.L.); zhangcheng605@163.com (J.X.); 21710036@stu.ahau.edu.cn (X.H.); machendong513@126.com (Y.L.); guoxing0405@126.com (X.G.)

**Keywords:** Wannan male chickens, feather follicle density, mRNA expression profiles, weighted gene co-expression network analysis

## Abstract

**Simple Summary:**

To study the molecular mechanism of chicken feather follicle density, weighted gene co-expression network analysis (WGCNA) and transcriptome analysis were established based on skin transcriptome data from Wannan male chickens. The results showed that *FOXM1*, *GTSE1*, *MELK*, *CDK1*, *ECT2*, and *NEK2* may be involved in regulating the development of feather follicle density. This study promotes our understanding of the molecular mechanism of feather follicle density.

**Abstract:**

Feather follicle density plays an important role in appealing to consumers’ first impressions when making purchasing decisions. However, the molecular network that contributes to this trait remains largely unknown. The aim of this study was to perform transcriptome and weighted gene co-expression network analyses to determine the candidate genes relating to feather follicle density in Wannan male chickens. In total, five hundred one-day-old Wannan male chickens were kept in a conventional cage system. Feather follicle density was recorded for each bird at 12 weeks of age. At 12 weeks, fifteen skin tissue samples were selected for weighted gene co-expression network analysis, of which six skin tissue samples (three birds in the H group and three birds in the L group) were selected for transcriptome analysis. The results showed that, in total, 95 DEGs were identified, and 56 genes were upregulated and 39 genes were downregulated in the high-feather-follicle-density group when compared with the low-feather-follicle-density group. Thirteen co-expression gene modules were identified. The red module was highly significantly negatively correlated with feather follicle density (*p* < 0.01), with a significant negative correlation coefficient of −0.72. In total, 103 hub genes from the red module were screened. Upon comparing the 103 hub genes with differentially expressed genes (DEGs), it was observed that 13 genes were common to both sets, including *MELK*, *GTSE1*, *CDK1*, *HMMR*, and *CENPE*. From the red module, *FOXM1*, *GTSE1*, *MELK*, *CDK1*, *ECT2*, and *NEK2* were selected as the most important genes. These genes were enriched in the DNA binding pathway, the heterocyclic compound binding pathway, the cell cycle pathway, and the oocyte meiosis pathway. This study suggests that *FOXM1*, *GTSE1*, *MELK*, *CDK1*, *ECT2*, and *NEK2* may be involved in regulating the development of feather follicle density in Wannan male chickens. The results of this study reveal the genetic structure and molecular regulatory network of feather follicle density in Wannan male chickens, and provide a basis for further elucidating the genetic regulatory mechanism and identifying molecular markers with breeding value.

## 1. Introduction

The feather is a complex epidermal organ with great diversity in shape, size, and pigmentation, which depends on the regulation of gene expression and the assembly of protein components [1]. For example, the *EDMTFH* gene is involved in feather differentiation and development [2]. The *EDDM* gene promotes the evolution of the most complex molecular structures in feathers [3]. HBS1 keratin is thought to be the building block of feathers [4]. In avian species, the primary function of feathers is to maintain body temperature and be used for flight, communication, and heat dissipation [5]. Feathers grow from the feather follicle, which consists of the follicle sheath (outer root sheath and inner root sheath), the feather barb ridges, and the collar bulge [6]. Generally, the development of feather follicles is characterized by two distinct phases: the growth phase and the resting phase. The quantity and placement of these follicles are determined during the embryonic stage, while their type or size may evolve throughout the developmental process [7]. In chickens, the feather follicles are arranged in clusters called bundles [8], and each bundle is a continuous set of feathers with regular spacing between initially adjacent feathers [9]. Feather follicle density is one of the most important economic traits in chickens, and is associated with the space between feather follicles, which is regulated by the distribution of initiation sites. Feather follicle density affects the appearance, carcass and energy efficiency of chickens, and has a significant impact on the economics of poultry production. Yuan et al. found that the marketing age to 90 days enhances the quality of carcass characteristics such as low feather follicle density [10]. Ji et al. reported that the feather follicle density of back skin was significantly higher than that of thigh skin [11]. In addition, chicken feathers account for approximately 5–10% of live weight, and represent a significant proportion of poultry waste [12]. Therefore, uncovering the genetic basis for feather follicle density traits is essential for future molecular breeding of this trait in chickens.

In modern commercial poultry production, the factors that affect feather follicle density are mainly the environment [13], feed [14], and genetics, the latter of which is one of the most important. In recent years, many studies have been conducted to explore the candidate genes and the molecular mechanisms underlying feather follicle density traits. For example, a polymorphic study showed that Wnt6a gene polymorphism was associated with feather follicle density in Wanxi white geese [15]. Based on microarray technology, the results showed that the Wnt signaling pathway (Wnt), the fibroblast growth factor signaling pathway (FGF), the mitogen-activated protein kinase signaling pathway (MAPK), the sonic hedgehog signaling pathway (SHH), and the bone morphogenetic protein signaling pathway (BMP) were involved in the regulation of feather follicle density in various chicken skin segments [11]. The GWAS analysis results showed that 146 SNP markers were identified as being related to the density of contour feathers. Several candidate genes, including *SUCLA2*, *DNAJC15*, *DHRS12*, *MLNR*, and *RB1*, were identified as potentially participating in the genetic control of chicken contour hair density [16]. Although many studies have reported the molecular genetic mechanism of feather follicle density traits, the regulatory network of feather follicle density remains largely unknown.

China is the world’s second largest producer and consumer of broiler chickens, and yellow-feathered chickens account for about 50% of China’s chicken production, with an annual output of more than 4 billion birds [17]. In recent years, with live bird markets closed in China, carcass appearance has become crucial in chicken sales. Therefore, it is of great significance to study the feather follicle characteristics of yellow-feathered chickens. As a carcass appearance trait, feather follicle density is important in appealing to consumers’ first impressions when making purchasing decisions. The Wannan chicken is a medium-size, slow-growing, and meat-and-egg dual-purpose local breed of Chinese chicken, and it has been listed as an indigenous poultry variety resource since 2003 [18]. Therefore, this study used Wannan chickens as a model to study and improve our understanding of feather follicle density development. In recent studies, transcriptomics has been used to discover differentially expressed genes related to target traits from a large amount of genomic information in chickens, thereby revealing the inherent relationship between gene expression and important economic traits [19,20]. Weighted gene co-expression network analysis (WGCNA) combines phenotypic data analysis of chicken transcriptome profiles and is used to find key module and hub gene networks [21,22]. Thus, the primary aim of this study was to conduct a transcriptome and weighted gene co-expression network analysis (WGCNA) to assess feather follicle density in Wannan male chickens.

## 2. Materials and Methods

### 2.1. Animals and Housing

In total, 500 1-day-old Wannan male chickens with similar body weight were provided with the same environmental and feeding regime. The ambient temperature was originally maintained at 32 to 34 °C at 1 week of age and then lowered by 2 °C per week until the temperature was about 20 °C. The light time was based on the stage of development (0 to 1 week: light time, 23 L/1 D; 1 to 2 weeks: light time, 20 L/4 D; 2 to 3 weeks: light time, 18 L/6 D; 3 to 4 weeks: light time, 16 L/8 D; 4 to 12 weeks: light time, 12 L/12 D). All chickens were kept in a conventional and identical cage system for 1 to 12 weeks. Birds had ad libitum access to water and a pellet diet appropriate for their stage of development (1 to 3 weeks: CP, 20.5% and ME, 12.59 MJ/kg; 4 to 7 weeks: CP, 18.5% and ME, 12.98 MJ/kg; and 8 to 12 weeks: CP, 17.0% and ME, 11.75 MJ/kg) [23].

### 2.2. Feather Follicle Density Data Collection

For each bird, feather follicle density was recorded at 12 weeks of age. A 2 × 2 cm^2^ sample of left and right back skin tissue was used to measure the feather follicle density, which was used to count the number of feather follicles [11]. Back skin was divided into two halves in the dorsal axis. Feather follicle density was found via the measurement of two typical locations on the left and right back skin only of each chicken, and then averaging these values. Based on feather follicle density, the top 30 male chickens with the highest feather follicle density were selected to make up the high-feather-follicle-density (H) group, and the bottom 30 male chickens with the lowest feather follicle density were selected to make up the low-feather-follicle-density (L) group.

### 2.3. RNA-seq Datasets

In total, 15 male chickens with a similar average body weight were selected to be slaughtered after fasting for 12 h, and then euthanized; bloodletting took place after electroshock (7 birds in the H group, and 8 birds in the L group). The back skin was sampled immediately after slaughter. Fifteen chickens’ right back skin from the same location was sampled at 12 weeks of age, immediately frozen in liquid nitrogen, and stored at −80 °C before RNA isolation. The TRIzol method (Invitrogen, Carlsbad, CA, USA) was used to extract total RNA from fifteen skin tissue samples. RNA quality was determined using the electrophoresis method and a NanoDrop spectrophotometer 2000 (Thermo Scientific, Wilmington, DE, USA). RNA-seq transcriptome libraries were prepared using a TruSeqTM RNA sample preparation kit from Illumina (Illumina Inc, San Diego, CA, USA) according to the manufacturer’s recommendations. Paired-end libraries were sequenced by Illumina NovaSeq 6000 sequencing (150 bp × 2, Shanghai BIOZERON Co., Ltd., Shanghai, China). 

### 2.4. RNA-seq Data Analysis

The discarding of adapters and low-quality reads was performed using Cutadaptoor and Btrim [24], with the parameters “-s -a 20 -q”. Then, clean reads were separately aligned to reference genome (Galgal 7.0) with orientation mode by using hisat2 (v 2.1.0) [25]. This software was used to map with default parameters. SAMtools (https://samtools.sourceforge.net/, accessed on 20 December 2023) was used to sort and index the bam files [26]. The Python (v 3.9.2) script htseq-count was used to count the number of reads for each gene [27]. Six sequencing datasets (3 birds in the H group and 3 birds in the L group) were utilized to analyze differential gene expression of feather follicle density. The DESeq2 R package (v 4.2.2) was used to calculate differential gene expression, with a *p* value < 0.05 and |log2fold-change| > 1 used as the significance threshold.

### 2.5. Weighted Gene Co-Expression Network Analysis

Fifteen sequencing datasets were utilized to analyze the weighted gene co-expression network. The DESeq2 [28] package (v 4.2.2) was used to perform gene expression level normalization with the “varianceStabilizing Transformation” function. We applied the WGCNA package tool within R software (v 1.7.1) to construct gene co-expression networks [29]. First, we calculated the Pearson correlation between genes, and constructed a gene co-expression correlation matrix. Subsequently, we selected the optimal soft threshold based on the criterion of approximate scale-free topology (β = 1 to 20), and generated a weighted adjacency matrix. Furthermore, module detection was performed using the cutreeDynamamic function with the parameters “minModuleSize = 30, deepSplit = 2, pamRespectsDendro = F”, and cutHeight = 0.3 was used to merge modules. The phenotypic data of feather follicle density and gene modules were quantified using the Pearson correlation and measured by correlation coefficient |R| ≥ 0.51, and *p* < 0.05 represented significant consensus modules. In addition, the gene significance (GS) module was used to quantitatively analyze the relationship between genes and traits. Then, the correlation analysis between GS and module members (MM) was performed, and the genes with GS > 0.2 and MM > 0.8 were identified as the hub genes in the trait specific module. Finally, the gene regulatory network in the key module was drawn using Cytoscape software (v 3.9.1) [30].

### 2.6. Functional Enrichment Analysis of DEGs and Hub Genes

The online annotation tool g: Profiler [31] was used to perform GO and KEGG pathway enrichment analyses of differentially expressed genes (DEGs) and hub genes of significant modules.

### 2.7. Real-Time Fluorescence Quantitative PCR

Eight right back skin tissue samples were randomly selected from the H and L groups for qPCR analysis. Twelve DEGs were selected for qPCR. Specific primers for gene amplification are shown in Supplementary Table S1 and were designed using Primer 3 Input. Total RNA was reverse-transcribed using a Fast Quant RT Kit (TIANGEN BIOTECH (Beijing) Co., Ltd., Beijing, China according to the manufacturer’s instructions. SuperReal PreMix SYBR Green (TIANGEN BIOTECH (Beijing) Co., Ltd., Beijing, China) was applied to perform the qPCR reaction with an ABI Prism 7500 instrument (Applied Biosystems, Carlsbad, CA, USA). Relative gene expression levels were determined by using the 2^–ΔΔCt^ method with the *GAPDH* gene for normalization [32].

### 2.8. Hub Genes Expression Analysis

In total, 60 Huaibei male chickens were selected. The feather follicle density weas recorded at 120 days of age. Based on the feather follicle density, the top 5 male chickens with the highest feather follicle density were selected to make up the high-feather-follicle-density (H) group and the bottom 5 male chickens with the lowest feather follicle density were selected to make up the low-feather-follicle-density (L) group. To verify whether the hub genes were associated with the feather follicle density trait, 10 right back skin tissue samples from Huaibei chickens (5 birds in the H group and 5 birds in the L group) were used for RNA-seq analysis. *FOXM1*, *GTSE1*, *MELK*, *CDK1*, *ECT2*, and *NEK2* were used for qPCR analysis. Specific primers for gene amplification are shown in Supplementary Table S1, and were designed using Primer 3 Input. Total RNA was reverse transcribed using a Fast Quant RT Kit (TIANGEN BIOTECH (Beijing) Co., Ltd., Beijing, China) according to the manufacturer’s instructions. qPCR analysis was performed on the ABI Prism 7500 instrument (Applied Biosystems, Carlsbad, CA, USA) using SuperReal PreMix SYBR Green (TIANGEN BIOTECH (Beijing) Co., Ltd., Beijing, China). Relative gene expression levels were determined by using the 2^–ΔΔCt^ method with the *GAPDH* gene for normalization [32].

### 2.9. Statistical Analyses

The feather follicle density was analyzed and hub gene expression differences between Wannan chickens and Huaibei chickens were identified by using an independent samples T-test with SPSS version 19.0 software (SPSS Inc., Chicago, IL, USA). Data are expressed as mean ± SD. The expression of back skin of low feather follicle density was set as the control group in the DEG validation and hub genes expression analysis. The threshold for significance was set at *p* < 0.05.

## 3. Results

### 3.1. Feather Follicle Density 

The feather follicle density of Wannan male chickens at 12 weeks of age is shown in Table 1. The feather follicle density was higher in the H group compared with the L group (*p* < 0.01). The average feather follicle density is shown in Table S2. The feather follicle density is 3.88.

### 3.2. Sequencing Data Evaluation

The transcriptome data evaluation is shown in Table S3. Fifteen cDNA libraries of 6 GB each were constructed from the total RNA of back skin samples collected from 15 Wannan male chickens. After quality control, clean data were mapped to the chicken reference genome (GRCg7a). The proportions of Q20 and Q30 bases were above 95.85% and 92.34%, respectively. The GC content of 15 samples ranged from 48.95% to 55.01%, and the statistical reading rate of the mapping was 77.66~90.45%.

### 3.3. mRNA Expression Profiling of Feather Follicle Density

**Figure 1:**
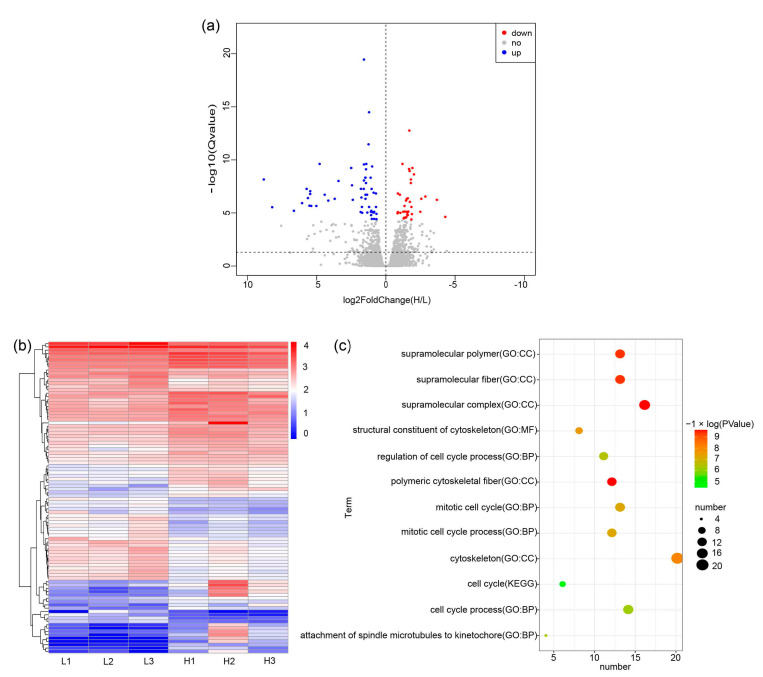
Identification of DEGs in the H and L groups. (**a**) Volcano plots of DEGs in the H and L groups; red denotes significantly downregulated and blue denotes significantly upregulated genes in the H group when compared with the L group. (**b**) Heatmap of DEGs in the H and L groups. H, high feather follicle density; L, low feather follicle density. (**c**) Enriched KEGG and GO pathways for differentially expressed genes (DEGs) identified between H and L groups. H, high feather follicle density; L, low feather follicle density.

Six total RNA samples of skin tissue samples were of high quality and used to construct sequencing libraries. Six cDNA libraries of 6 GB each were constructed from the total RNA of skin tissue samples collected from three birds in the H group and three in the L group. After quality control, the clean data were mapped to the chicken reference genome (Galgal7.0). In total, 14,909 genes were detected in the birds’ skin tissues (Figure 1a,b). Differentially expressed genes (DEGs) were screened by using the DESeq2 package. In total, 95 DEGs were identified, as shown in Figure 1a and Supplementary Table S4. Of these DEGs, 56 genes were upregulated, and 39 genes were downregulated in the high-feather-follicle-density group when compared with the low-feather-follicle-density group (Figure 1 and Table S3). These DEGs were enriched in 66 GO terms and 1 KEGG term (Figure 1c and Table S5), for example, cell cycle, structural constituent of cytoskeleton, mitotic cell cycle process, cell cycle process, and supramolecular polymer.

### 3.4. WGCNA for Feather Follicle Density

**Figure 2:**
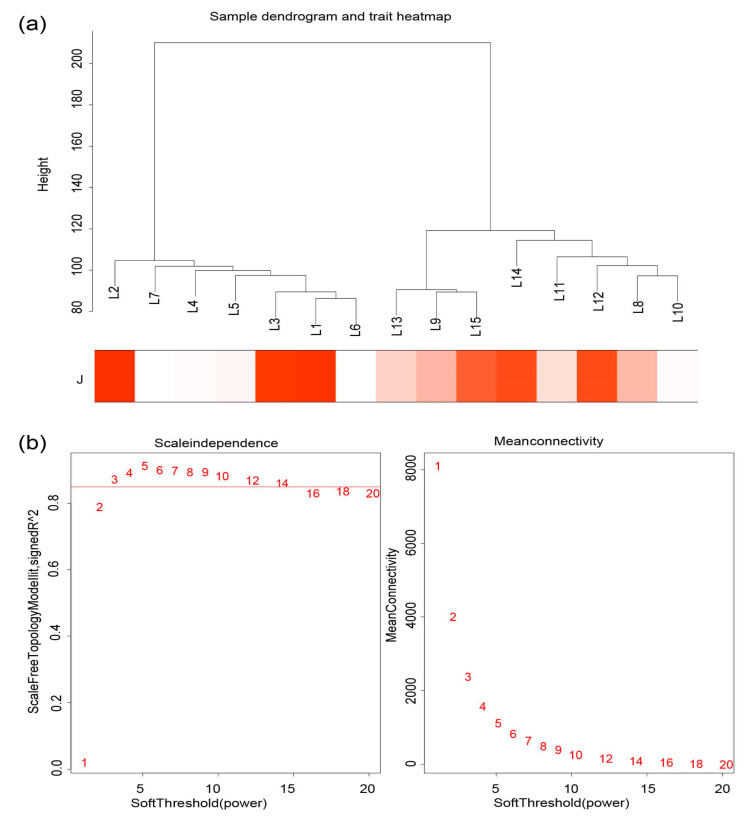
WGCNA of gene expression profile with feather follicle density in Wannan male chicken skin tissue at 12 weeks. (**a**) Heat map of sample clustering and traits; J, feather follicle density; The color red indicates the correlation between phenotypic data and gene expression. The darker the shade of red, the stronger the correlation. (**b**) Scale-free topology model fit, and gene mean connectivity under different soft threshold powers.

Based on the transcriptome data of the Wannan male chicken skin tissue at 12 weeks, in total, 24,559 genes were obtained for WGCNA (Table S6). A cluster analysis of skin tissue samples and feather follicle density traits is shown in Figure 2a. The feather follicle density varied greatly in different samples. Keeping to the scale-free topology criterion, β = 3 was considered in this study (Figure 2b). Based on the gene clustering tree analysis, a total of 13 modules, including midnight blue, black, yellow, red, and salmon were identified, and the number of genes in the five mentioned modules was 118, 230, 304, 257 and 313, respectively (Figure S1). The gray module was composed of genes that did not belong to any specific module.

### 3.5. Hub Genes Related to Feather Follicle Density

**Figure 3:**
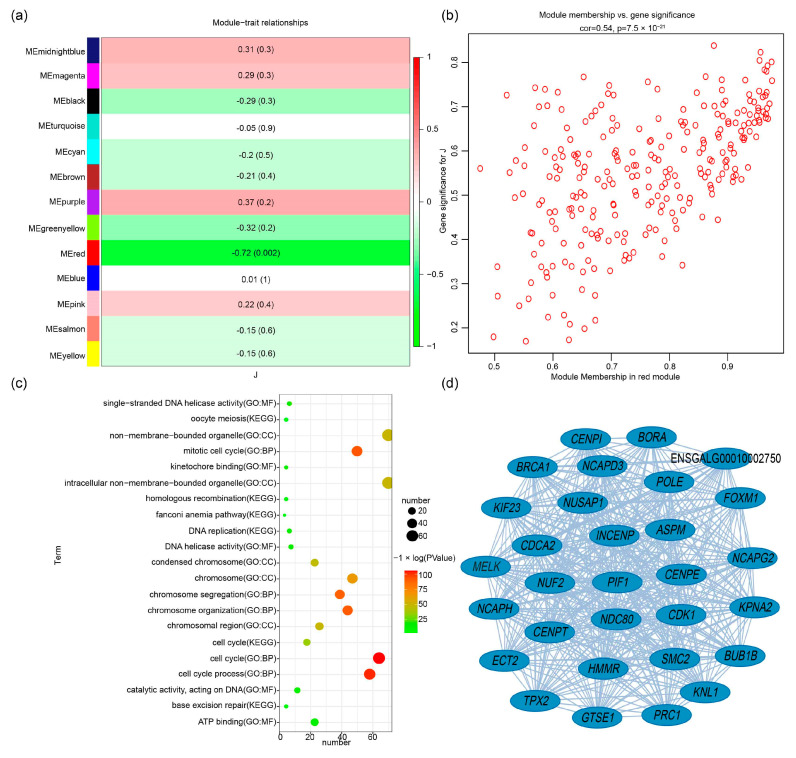
WGCNA of gene expression profiles with feather follicle density in Wannan male chicken skin tissue at 12 weeks. (**a**) Analysis of the correlation between different module genes and phenotypes; (**b**) GS and MM analysis for density; (**c**) enriched KEGG and GO pathways for hub genes in the red modules; (**d**) interaction network of the hub genes in the red modules. J, feather follicle density.

Among the 13 modules, the red module was highly significantly negatively correlated with feather follicle density (*p* < 0.01), with a significant negative correlation coefficient of −0.72 (Figure 3a). After identifying the important modules, the red module had 257 genes (Table S7). The hub genes in the red module were mined using GS and MM, and 103 hub genes were obtained from the red module (Table S8). The correlation between GS and MM was 0.54 (*p* < 0.01) (Figure 3b). Functional enrichment analysis shows that these hub genes on the red module were enriched in 419 GO terms such as structural constituent of cytoskeleton, mitotic cell cycle process, supramolecular complex, polymeric cytoskeletal fiber, and structural constituent of cytoskeleton. These hub genes on the red module were enriched in six KEGG terms, namely cell cycle, DNA replication, base excision repair, homologous recombination, oocyte meiosis, and the fanconi anemia pathway (Figure 3c and Table S9). Cytoscape software (v3.9.1) was used to generate the interaction network diagrams for the top 30 hub genes in the red module, as shown in Figure 3d. Then, 103 hub genes in the red module were compared with RNA-seq differentially expressed genes in the skin tissue of Wannan male chickens in our previous study (Table S10), and a total of 13 hub genes were identified: maternal embryonic leucine zipper kinase (*MELK*), G2 and S phase-expressed 1 (*GTSE1*), cyclin-dependent kinase 1 (*CDK1*), hyaluronan mediated motility receptor (*HMMR*), centromere protein E (*CENPE*), epithelial cell transforming 2 (*ECT2*), BUB1 mitotic checkpoint serine/threonine kinase B (*BUB1B*), forkhead box M1 (*FOXM1*), telomeric repeat binding factor 1 (*TERF1*), NIMA related kinase 2 (*NEK2*), minichromosome maintenance complex component 3 (*MCM3*), cell division cycle associated 3 (*CDCA3*), and a new gene.

### 3.6. Quantitative Real-Time PCR Validation

**Figure 4:**
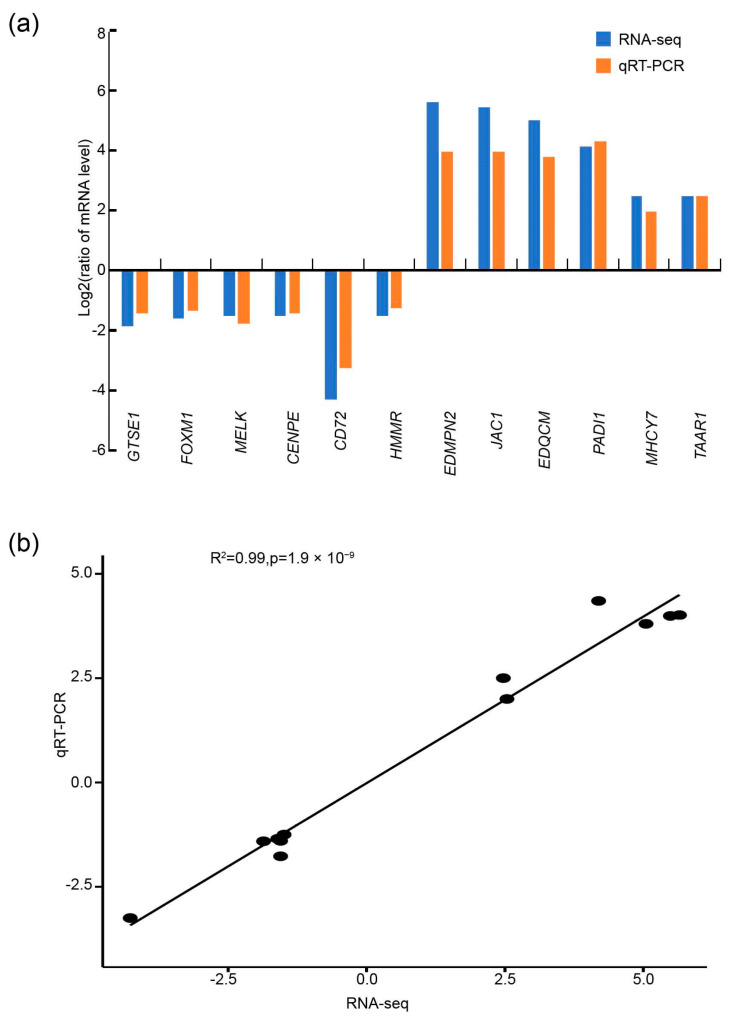
(**a**) Comparisons between qRT-PCR and RNA-seq measurements of the expression abundance of 10 random differentially expressed genes; (**b**) correlation analysis of the values between RNA-seq and qPCR results.

The analysis of the expression levels of the DEGs are shown in Figure 4. All selected DEGs showed concordant expression patterns in the RNA-seq and qPCR results. The results of qPCR were highly correlated with the results of RNA-seq analysis (R^2^ = 0.99).

### 3.7. Hub Genes Expression

The feather follicle density of Huaibei male chickens at 120 days of age is shown in Table S11. The feather follicle density was higher in the H group compared with the L group (*p* < 0.01). The analysis of the expression levels of the hub genes are shown in Table S12 The results showed that the expression levels of *FOXM1*, *GTSE1*, *MELK*, *CDK1*, *ECT2*, and *NEK2* were lower in the high-feather-follicle-density group compared with the low-feather-follicle-density group (*p* < 0.01).

## 4. Discussion

It is of important to study the feather follicle density of chickens for improving the breed selection and economic benefits. In recent years, there have been many reports on the regulatory mechanism of the growth and development of chicken feather follicles, which is a complex multistep developmental process [33,34,35]. While many genes have been found to be associated with feather follicle development, the gene network associated with feather follicle density has not been clearly defined. WGCNA is an excellent bioinformatics tool for in-depth assessment of changes in feather follicle density gene expression at the phenotypic level [36]. In this study, key co-expression biological functional blocks and key genes that may play a key role in feather follicle density were identified by integrating phenotype and transcriptome data. In our study, through gene cluster tree analysis, we divided all co-expressed genes into 13 co-expressed biological functional modules. Of the 13 main co-expression biological functional modules, the red module had the most significant relationship with feather follicle density. In total, 103 hub genes were identified. To further understand the importance of functional modules in feather follicle density, KEGG and GO functional enrichment analyses were subsequently conducted, which mainly enriched some biological processes such as cell cycle, DNA replication, base excision repair, homologous recombination, oocyte meiosis, and the fanconi anemia pathway. Furthermore, it is critical to find the hub gene among the many genes in the most important module. Afterwards, we found that among the most important functional module (red module), *FOXM1*, *GTSE1*, *MELK*, *CDK1*, *ECT2*, and *NEK2* were the most important genes.

At present, studies of feather follicle traits are receiving a lot of attention. There have been many related reports on the regulation mechanism of poultry skin feather follicle growth and development. The Wnt signaling pathway encompasses over 19 members of the family, including both canonical and non-canonical Wnt pathways. [37]. The Wnt/β-catenin signaling pathway is involved in the regulation of feather follicle development and feather growth during chick embryonic development [38]. The Wnt signaling pathway is involved in the development of feather follicle density and diameter in embryonic chickens [39]. We found that these hub genes may affect pathways involved in feather follicle density. The *GTSE1* (G2 and S phase-expressed1) gene is located on chromosome 22q13.2-q13.3 [40], and *GTSE1* knockdown results in inactivation of Wnt/β-catenin signaling [41]. *MELK* (maternal embryonic leucine zipper kinase) has remarkable sequence identity with Snf1/AMPK family kinases [42]. Knockout of *MELK* is found to affect the expression of genes related to the Notch/Wnt signaling pathway [43]. *CDK1* (cyclin-dependent kinase 1) is one of the cyclin-dependent kinases and serine/threonine kinases [44] whose expression of *CDK1* affects the Wnt signaling pathway [45]. The *ECT2* (epithelial cell transforming 2) gene is a guanine nucleotide exchange factor of the Rho family of GTPases [46]. The *ECT2* gene is enriched in the Wnt signaling pathway as a hub gene in duck feathers [47]. The *NEK2* (never in mitosis gene A (NIMA)-related kinase 2) gene is a CIN gene located at 1q32.2, is the most significant, and is a serine/threonine kinase [48]. Knockdown of *NEK2* inhibits activation of the Wnt1/β-catenin signaling pathway [49]. The Wnt/β-catenin pathway is the downstream pathway of *NEK2,* which promotes cell proliferation, migration and invasion by activating the Wnt/β-catenin pathway [50]. So, the *GTSE1*, *MELK*, *CDK1*, *ECT2*, and *NEK2* genes may be involved in the molecular regulation of feather follicle density.

The cell cycle is a series of events in which cells replicate and divide and regulate cell growth, proliferation, development and death [51,52]. The cell cycle comprises several distinct phases: the G1 phase (resting), S phase (synthesis), G2 phase (interphase), and M phase (mitosis) [53]. The regulation of the cell cycle is a complex process orchestrated by an intricate network of interactions involving proteins, enzymes, cytokines, and various cell cycle signaling pathways, and is crucial for the proliferation, growth, and repair of cells [54]. The cell cycle has important implications for human hair follicle (HF) biology, and active cell cycles promote healthy hair growth [55]. Thus, the cell cycle may affect the development of feather follicle density in chickens.

The JAK/STAT signaling pathway is the Janus kinase–signal transducer and activator of transcription signaling pathway, in which STAT is phosphorylated by JAK, dimerized, and then transported through the nuclear membrane to the nucleus to regulate the expression of related genes [56]. The JAK/STAT signaling pathway is involved in various bodily functions and involved in a number of important biological processes, including cell proliferation, differentiation, apoptosis, immune regulation, and hematopoiesis [57]. The Janus kinase–signal transducer and activator of transcription (JAK/STAT) signaling pathway has been found to regulate feather follicle morphogenesis, feather follicle development, and feather follicle growth [58]. The *FOXM1* (forkhead box M1) gene is a member of the forkhead box (FOX) family of transcription factors [59], and the JAK/STAT signaling pathway could be negatively affected by the *FOXM1* gene [60,61]. A study has found that SPHK1 inhibition with SKI-178 by *CDK1* leads to decreased JAK/STAT signaling [62]. Embelin downregulates the expression of *CDK1* and modulats the JAK/STAT pathway, which is related to cell growth and apoptosis [63]. The *CDK1* gene was significantly enriched in the JAK/STAT signaling pathway [64]. Therefore, the *FOXM1* and *CDK1* genes may influence the development of feather follicle density.

The fibroblast growth factor (FGF) family is divided into signaling proteins secreted by signaling receptor tyrosine kinases (secreted FGF) and intracellular non-signaling proteins (intracellular FGFs (iFGFs)) that act as voltage-gated sodium channels and other molecular cofactors [65]. In the early stages of embryonic development, FGFs are involved in cell proliferation and morphogenesis. In developing chicken skin, FGFs can initiate feather substrate formation and enhance feather density [66]. The sprouty/FGF signaling pathway has been found to regulate feather follicle morphogenesis, feather follicle development, and feather follicle growth [67]. Negative regulation of the fibroblast growth factor signaling pathway could affect *FOXM1* transcription factor expression and subsequent cell cycle progression [68]. Cyclin-dependent kinase 1 (*CDK1*) is responsible for the fibroblast growth factor signaling pathway that affects cell proliferation [69]. Accordingly, these studies suggest that *FOXM1*, *GTSE1*, *MELK*, *CDK1*, *ECT2*, and *NEK2* may regulate the development of feather follicle density through biological processes such as the Wnt signaling pathway, the Janus kinase–signal transducer and activator of transcription (JAK/STAT) signaling pathway, and the fibroblast growth factor (FGF) signaling pathway.

## 5. Conclusions

Transcriptome analysis of different feather follicle densities of Wannan male chickens, combined with differential expression analysis and WGCNA, helped to identify multiple potential candidate genes regulating feather follicle density. One specific co-expressed biological functional module and six key regulatory genes, namely, *FOXM1*, *GTSE1*, *MELK*, *CDK1*, *ECT2*, and *NEK2*, were identified for their effect on feather follicle density in chicken skin tissue. These results reveal the genetic structure and molecular regulatory network of feather follicle density traits in Wannan male chickens, and provide a basis for further elucidating the genetic regulatory mechanism and identifying molecular markers with breeding value.

## Figures and Tables

**Table 1:** Feather follicle density of Wannan male chickens at 12 weeks of age.

Group (*n* = 30)	Mean
H (piece/cm^2^)	4.95 ± 0.19 ^A^
L (piece/cm^2^)	3.27 ± 0.04 ^B^

Different superscript letters A and B in the same row indicate extremely significantly difference (*p* < 0.01). H, high feather follicle density; L, low feather follicle density.

## Data Availability

The RNA-seq data from this study were deposited in the NCBI (https://www.ncbi.nlm.nih.gov/), and the accession number is PRJNA1021778 (accessed on 26 September 2023).

## Supplementary Materials

The following supporting information can be downloaded at: https://www.mdpi.com/article/10.3390/ani14010173/s1. The data presented in this study are contained within the article or Supplementary Materials. Figure S1: WGCNA analysis of gene expression profile with feather follicle density in Wannan male chickens’ skin tissue at 12 weeks. The cluster dendrogram constructs the gene modules and module merging; Table S1: Primers for qRT-PCR; Table S2: Feather follicle density of Wannan male chickens at 12 weeks of age; Table S3: Summary of RNA-seq data; Table S4: Detailed information on DEGs between H and L groups in chickens; Table S5: Enriched GO and KEGG pathways for DEGs identified between H and L groups; Table S6: Gene list analyzed by WGCNA; Table S7: Gene list of the red module; Table S8: List of hub genes indicated in the manuscript with a red module; Table S9: Enriched GO and KEGG pathways for hub genes in the red module; Table S10: List of differentially expressed genes in skin of Wannan male chickens at 12 weeks and the 103 hub genes in the red module; Table S11: Feather follicle density of Huaibei male chickens at 120 days of age; Table S12: Hub gene expression level.
