# Supplementary material for: Transcriptome and Weighted Gene Co-Expression Network Analysis for Feather Follicle Density in a Chinese Indigenous Breed
Source: Animals (Basel). 2024 Jan 4;14(1):173. doi: 10.3390/ani14010173 (PMC10778273; doi:10.3390/ani14010173)
Supplement: Supplementary file 1 [file animals-14-00173-s001.zip › Supplementary Figure.pdf]

Supplementary Figure

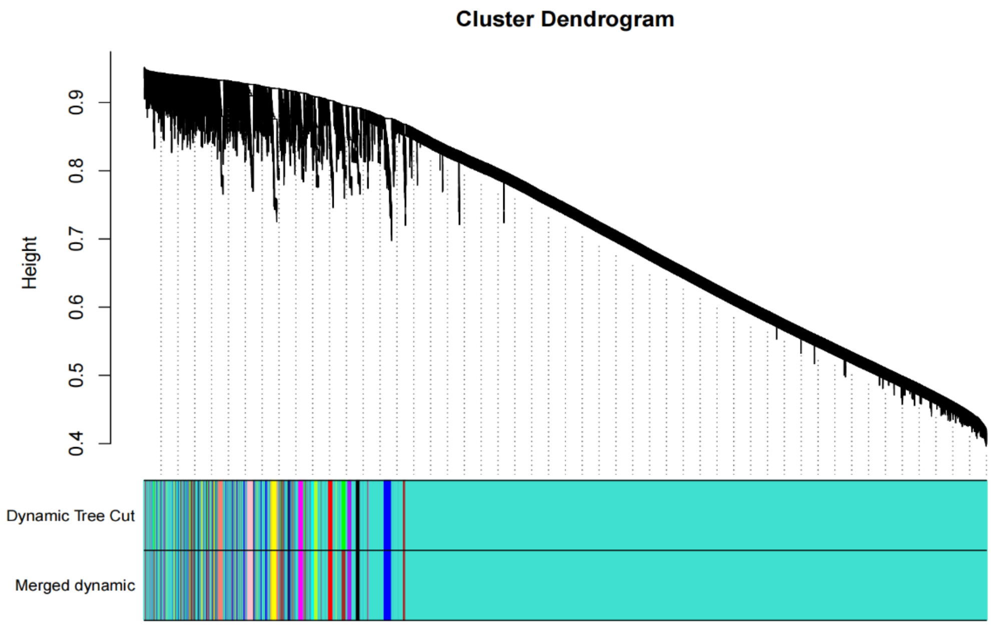

Figure S1. WGCNA analysis of gene expression profile with feather follicle density in Wannan male chickens' skin tissue at 12 weeks. The cluster dendrogram constructs the gene modules and module merging.
